# Bacterial Sentience Is Determined by the Stochastic, Chaotic, and Deterministic Behavior of Cytoplasmic Particles

**DOI:** 10.3390/cimb48080777

**Published:** 2026-07-30

**Authors:** Leon M. T. Dicks, Carolina Pohl, Alfred Botha

**Affiliations:** 1 Department of Microbiology, Stellenbosch University, Stellenbosch 7600, South Africa; abo@sun.ac.za; 2 Department of Microbiology and Biochemistry, University of the Free State, Bloemfontein 9300, South Africa; pohlch@ufs.ac.za

**Keywords:** bacterial sentience, stochastic behavior, chaotic behavior, deterministic behavior

## Abstract

Bacteria are constantly exposed to stress, which intensifies as cells age, nutrients are depleted, and metabolite levels change. As metabolic activity increases, the cytoplasm transitions from a glass-like to a more fluid state, supporting the stochastic (spontaneous) and chaotic (nonlinear and unpredictable) movement of particles. The rate at which suspended particles or those in vacuole-like “cages” move depends on the cytoplasm’s energized and fluidic state. Cells respond to stochastic and chaotic behavior by regulating gene transcription, translation, and post-translational modifications. These stochastic and chaotic reactions generate a liquid–liquid phase separation (LLPS), causing particles to separate. This produces a dynamic force that drives cytoplasmic “turnover”. Internal and external physicochemical changes are monitored by chemoreceptors on the cell surface and embedded in the cell membrane, which activate transcriptional regulators to control gene expression, modulate enzymatic fluctuations, and regulate post-translational modifications. Sentience may also arise from the quantum-like behavior of ions, electrons, neutrons, and protons (tunneling and entanglement) and from hyperstructures that drive complex enzymatic reactions. This is, however, a highly debated topic. We argue that bacteria are conscious and do not rely solely on phosphorylation states, as in two-component systems (TCSs), but also on other cytoplasmic dynamics. We provide several examples to support the argument. It is, however, important to note that bacterial consciousness cannot be compared to that of higher life forms with a central nervous system. We refer to bacteria’s awareness of their environment as sentience and define bacterial sentience as the ability to respond to external stimuli and to reactions within a dynamic cytoplasm, thereby transferring signals either directly or via signal transduction pathways to turn gene expression on or off. We also point out that stochastic/chaotic randomness keeps the cytoplasm in a permanently dynamic, flexible, and stochastic state, safeguarding the cell against sudden, unpredictable environmental changes.

## 1. Introduction

**Figure 1:**
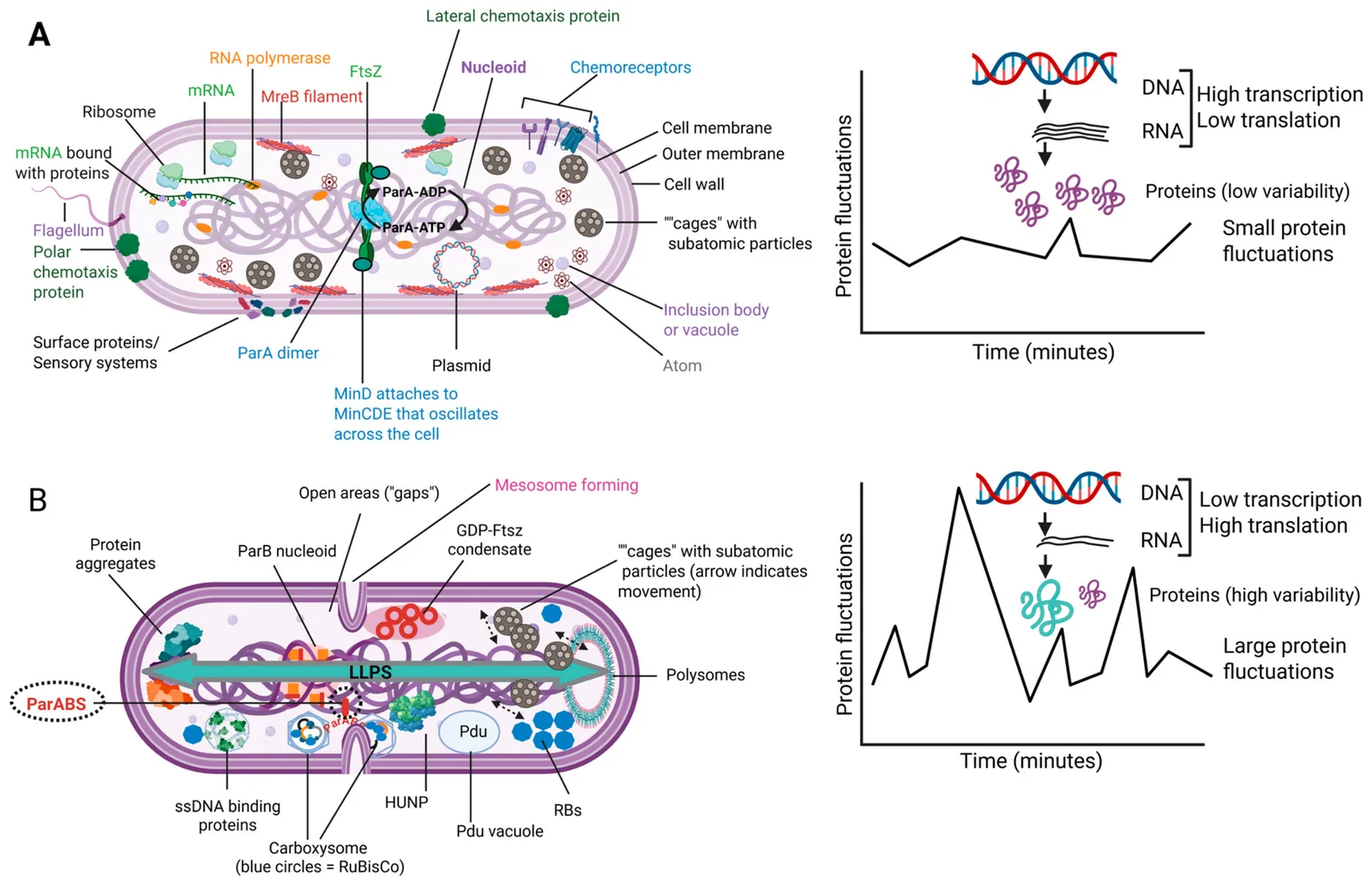
An illustration of stochastic, chaotic, and deterministic behavior of particles and structures in the cytoplasm of a bacterial cell (Gram-negative as an example). (**A**) **Left image**: The nucleoid, plasmids (if present), subatomic particles (e.g., electrons), atoms, molecules, proteins, and ribosomes float randomly in the cytoplasm. Enzymatic megacomplexes such as MreB filaments (actin homolog) and chemotaxis proteins attach to the cell membrane, the ParA protein dimer attaches to the genome, MinD (an ATPase) attaches to MinCDE that oscillates across the cell, and the FtsZ protein ensures the FtsZ ring (Z-ring) assembles in the middle of the cell. Molecules, atomic and subatomic particles reorganize and group into microcompartments (cages). **Right image**: At high transcription rates, fluctuation (variability) in protein levels is low. The same low protein fluctuations are seen in transcription without bursts (low cytoplasmic noise). (**B**) **Left image**: With an increase in metabolic processes, the cytoplasm becomes more fluidic. Particles and cages separate, forming open areas (gaps) in the cytoplasm. The movement of particles generates dynamic heterogeneity within the cytoplasm. The exchange between ADP and ATP in ParA (shown in (**A**)) “shifts” the genome towards opposite poles of the cell, forming a “wave”-like movement. RNA polymerases, ribonucleoprotein bodies (RBs), propanediol utilization (Pdu) microcompartments, heat-unstable nucleoid-associated proteins (HUNPs), carboxysomes, single-strand DNA (ssDNA) binding proteins, protein aggregates, and polysomes group into separated dense areas in the cytoplasm, resulting in a liquid–liquid phase separation (LLPS, indicated by a blue arrow). The carboxysomes are linked to the genome by the ParA/ParB system (ParABS). ParB binds to specific centromere-like DNA sequences known as *parS*a and organizes, compacts, and segregates genomic and plasmid DNA into daughter cells. RuBisCo = Ribulose-1,5-bisphosphate carboxylase/oxygenase. **Right image**: At low transcription rates and higher translation, gene expression is noisier and fluctuation (variability) in protein levels is higher. The same high protein fluctuations are seen with bursts in transcription, even when the same mean number of transcripts are produced (noisy cytoplasm). Created in BioRender. Dicks, L. (2026) https://BioRender.com/a7sy9uz.

The bacterial cytoplasm is a dynamic, crowded, fluid environment in which subatomic, atomic, and macromolecular particles move and interact (Figure 1). Because the bacterial cytoplasm lacks organelles, particles must self-organize to generate an energized state. In a resting (metabolically inactive) state, the cytoplasm appears solid and glass-like (Figure 1A) [1]. The cytoplasm may be described as a multi-part machine that reacts to external stimuli and maintains the cell membrane, cell wall, and extracellular structures such as capsules, flagella, fimbriae, pili, and conjugation tubes. The densely packed elements, such as genetic material, subatomic particles, ions, atoms, inorganic and organic molecules, proteins, ribosomes, enzymatic megacomplexes, granules, and microcompartments, contribute to the highly polydisperse, glass-like appearance of the cytoplasm (Figure 1A) [1]. Metabolic processes fluidize the cytoplasm, separating larger particles from smaller ones, allowing larger particles to break free from local traps and move efficiently through the cell [1]. Large and small particles in the cytoplasm interact directly or group in vacuole-like structures called “cages” (Figure 1A) [1]. Examples of particles enclosed in cages include protein condensates, aggregates, and agglomerates; enzyme condensates such as ribulose-1,5-bisphosphate carboxylase/oxygenase (RuBisCO); DNA and DNA-binding proteins from starved cells; polyphosphate biopolymers; ribonucleoprotein bodies; and glycogen [2]. In a dynamic (metabolically active) state, the cytoplasm becomes less glassy and more fluidic (Figure 1B) [3,4]. Toward the end of the cell’s life, heat-unstable nucleoid protein (HUNP, Figure 1B) relaxes the highly compacted nucleoid [2]. Particles and cages rearrange, and open areas (“gaps”) form in the cytoplasm [1]. At this stage, the mesosome forms (Figure 1B) and the nucleoid divides [5], followed by binary fission.

As cells age, these “pockets” rearrange and coalesce, resulting in relatively large open areas (gaps) in the cytoplasm [1]. Due to differences in the size and mobility of these cages, particles become irregularly distributed throughout the cytoplasm, and the cytoplasm’s fluidity decreases over time [1]. Faster-moving cages are displaced more quickly from neighboring macromolecules and occupy more cytoplasmic space [1]. Slow-moving cages have a lower probability of escaping macromolecules and are easily encaged, thereby being confined to a specific area of the cytoplasm [1]. The difference in the speed at which cages move generates dynamic heterogeneity within the cytoplasm, described as the “turnover” of cytoplasmic content. Metabolically active cells must manage the “placement” (orientation) of particle cages and maintain their spatial arrangement to synchronize cellular reactions. Perhaps most important is the nucleoid’s spatial arrangement, as it must be equally divided during binary fission of the cell. On the other hand, the DNA of metabolically inactive cells is not protected by repair enzymes, and dormant cells must employ alternative mechanisms to protect their DNA until environmental conditions become more favorable. One way to protect the genome is through liquid-crystalline ordering, i.e., the formation of nanocrystalline and liquid-crystalline DNA arrangements [6]. Specialized DNA-binding proteins, such as Dps, condense the genome. The genome of dormant spores is protected by small, acid-soluble proteins (SASPs) [6].

The cytoplasm of metabolically active cells is described as “noisy” due to the exchange of molecules across the cell membrane, gene expression, protein synthesis, enzymatic reactions, and interactions among subatomic particles [7]. The behavior of cytoplasmic particles is stochastic (spontaneous) [8,9,10] or chaotic (nonlinear and unpredictable) [11]. The rate at which particles move depends on the fluidic and energized state of the cytoplasm [1]. According to Meng et al. [3], the collision of cages, depending on their size, can generate a force of 0.57 pN (piconewtons), which is sufficient to mobilize a protein of 10 nm and generate an active force of 0.4 pN. The physiological state of each cell varies at any given moment. Variations in the cytoplasmic fluidity of genetically identical bacteria may give rise to cells with different phenotypes [12,13].

Cytoplasmic dynamics (cytoplasmic turnover) remain poorly understood. Mathematical models provide some insight into the forces involved and how cytoplasmic fluidity arises [3,14,15,16,17]. Gene expression in *Escherichia coli* cells exposed to oxidative stress exhibits chaotic behavior, driven by a combination of stochastic and deterministic (predictable) particle movements [14]. Deterministic cytoplasmic behavior is driven by active metabolic energy (ATP), structural crowding, and phase separation. This allows the cell to predictably regulate internal transport and cell division [1]. Deterministic control is well demonstrated by the *Caulobacter crescentus* cell-cycle circuit, in which the master regulator *CtrA* is switched on and off in a fixed, reproducible sequence. Recent work [18] shows that this network is remarkably robust, even when the essential CckA signaling kinase is severely impaired. Compensatory mutations in the nitrogen regulatory protein *NtrC* reconfigure metabolism and gene expression to preserve reliable cell-cycle progression [18]. This robustness shows that, alongside the stochastic and chaotic dynamics highlighted elsewhere in this review, bacterial cytoplasmic behavior also includes tightly buffered, deterministic circuits that actively resist perturbation. We do not know how other stressors influence cytoplasmic turnover. It may be that the stressor determines whether the cytoplasm behaves stochastically, chaotically, or deterministically.

In this review, we discuss how stress influences the nonlinear (stochastic and chaotic) and linear (deterministic) behavior of the cytoplasm, and how these phenomena, in turn, affect bacterial sentience. We acknowledge that stressors affect bacteria differently and that responses vary among species; as a result, we provide a general overview.

## 2. Dynamics of a Stressed Cytoplasm

Bacteria are acutely aware of their environment, especially when stressed, and the cytoplasm is in a state of chaos. It stands to reason that bacterial sentience (environmental sensing) is influenced by the availability and type of nutrients, physiological conditions, and communication between cells via, e.g., autoinducers (AIs). Cells respond to stress and intracellular communication by regulating gene transcription, translation, and post-translational processes [19,20,21]. Cytoplasmic chaos may force cells to adopt a bet-hedging strategy, i.e., spreading risks to safeguard cells against the effects of stressors [22,23]. When stressed, the cytoplasm’s fluidic state changes. Liquid–liquid phase separation (LLPS, Figure 1B) enables the distribution and regrouping of particles and cages [2,24,25]. The rearrangement of particles and cages generates a dynamic force, leading to cytoplasm turnover. This puts the cytoplasm in a permanent chaotic or stochastic state. One may thus conclude that protein expression occurs in a stochastic and chaotic environment. The stochastic (random) metabolic behavior of stressed *E. coli* was described by Kiviet et al. [26]. Raj and van Oudenaarden [10], citing, amongst others, the work of Elowitz et al. [9], described randomness as multiple gene expressions within a single cell (e.g., the on- and off-expression of a specific gene), or fluctuations in the expression of two gene copies, due to variations in the numbers of RNA polymerases or ribosomes. “Bistable” (or multistable) states arise when small variations in gene expression prevent a cell from changing (“flip”) from one state to another. Large stochastic fluctuations in gene expression can induce a transition from one state to another.

Some molecules are encapsulated into microcompartments and are then translocated. *Salmonella enterica* sequesters the toxic aldehyde intermediate (propionaldehyde) generated during 1,2-propanediol catabolism to limit cytotoxicity [27]. *Salmonella enterica* and *Escherichia coli* convert ethanolamine into carbon and nitrogen in protein-bound microcompartments (metabolosomes) [28,29]. Cyanobacteria encapsulate CO_2_ in protein- and membrane-bound carboxysomes (Figure 1B) [30,31]. Carboxysomes are maintained in position by the ParA/ParB partitioning system (the ParABS system; Figure 1B). They attach to the nucleoid (Figure 1B), “pulling” it in opposite directions. Protein McdB attaches to the carboxysome, followed by the attachment of an ATPase (McdA) to the nucleoid (Figure 1B). As soon as the McdAB protein duplex forms, McdA hydrolyzes ATP, and the proteins are released from the DNA. This is referred to as the Brownian ratchet or “DNA relay” mechanism [32]. The randomly positioned McdA leads to the formation of a chemophoretic (thermodynamic) force, which may be described as carboxysomes “rushing towards” the highest concentrations of McdA on the nucleoid [33]. This prevents the clumping of carboxysomes [34].

In modeling a bacterial cell, Meng et al. [3] showed that the dynamics of the cytoplasm arise from a combination of metabolic processes. The magnitude of the active force (F) in the cytoplasm was determined to be 0.57 pN (pico-Newton) [3], deduced from the collision of small molecules (e.g., amino acids and ions) that are not in equilibrium. Meng et al. [3] estimated F as the thermal energy divided by a typical protein size of approximately 10 nm. Based on the equation *F* = *k_B_T*/*a*, where F is the force expressed in N, *k_B_* is the Boltzmann constant [≈1.38 × 10^−23^ J/K (Joules per Kelvin)], *T* is the temperature in K, and *a* is the size (in this case, 10 nm), a protein of 10 nm can be propelled by a force of 0.4 pN.

Despite seemingly chaotic conditions, key metabolic processes, such as nutrient uptake, energy production, and the synthesis of amino acids, lipids, and nucleotides, are characteristic of each species and remain conserved [35]. Post-translational modifications may be key to regulating enzymatic fluctuations [9,36]. This may reflect a trade-off between energy yield and protein cost, as observed in glycolysis [37]. Slight variations in how cells perceive stress may alter their phenotypic properties within the same population. This change in behavior, driven by metabolic specialization, confers fitness advantages, even under steady-state environmental conditions. This was illustrated with *E. coli* cultured with limited glucose and arabinose [38]. When the concentrations of the two sugars were increased, the same cells preferred glucose over arabinose, indicating that gene expression and sugar transport are controllable. Cells of *Bacillus subtilis* exposed to toxic levels of acetate (produced by the metabolism of glucose and malate) exhibited stochastic behavior and shut down metabolism and gene transcription [39]. Distinct gene expression profiles have also been reported in *Methylobacterium extorquens*, resulting in subpopulations that are more tolerant of toxic levels of formaldehyde [40].

Stochastic enzyme behavior can lead to random switching of promoters between transcriptionally active and inactive states and to DNA supercoiling [41]. This leads to fluctuations in mRNA and protein synthesis [42], an increase in protein/enzyme noise [43,44], and transcription, referred to as transcriptional bursting [45,46].

## 3. Sentience Derived from Chaos

**Figure 2:**
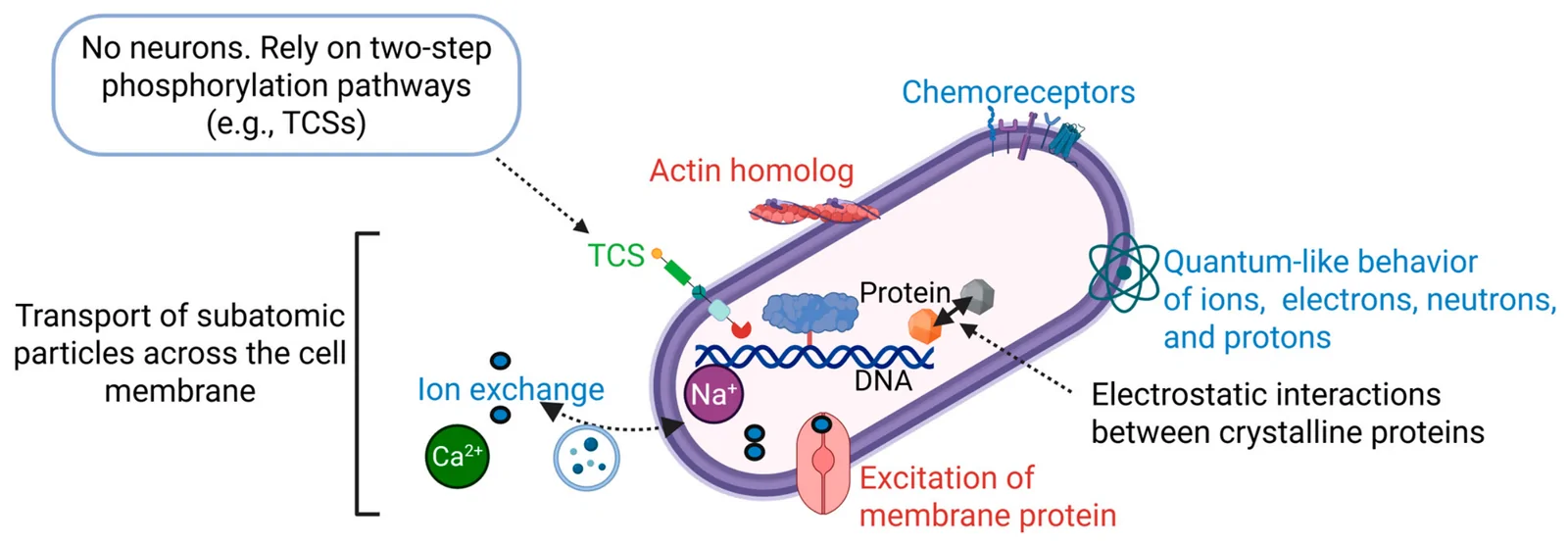
Bacteria do not have a nervous system and rely on mechanisms such as ion exchange, TCSs (two-component systems such as histidine kinase), actin homologs (e.g., mreB), chemoreceptors located in the cell membrane, quorum-sensing (QS) molecules such as auto-inducers, quantum mechanics (quanta), and membrane proteins to make decisions. Created in BioRender. Dicks, L. (2026) https://BioRender.com/5l7f261.

Robinson et al. [47] defined sentience as “feelings” or behavior based on “experiences,” and cognition as the ability to receive, process (learn), store, and act (decide) based on observations (e.g., of the environment) and memory (summarized in Figure 2). The authors argue that bacteria do not make choices, that decisions are determined by their current state, and that they rely on simple two-step phosphorylation pathways to regulate gene expression. For a bacterial cell to respond to environmental changes and maintain sentience, it is important to regulate the transport of subatomic particles across the cell membrane. This was concluded more than a decade ago, when Cook et al. [48] showed that the transport of cations across the cell membrane and the excitation of membrane carriers stimulate cells and, in turn, sentience. At about the same time, Gardiner [49] suggested that electrostatic interactions between crystalline proteins may generate endomembrane-based signaling. Sentience or cellular responsiveness may also arise from the quantum-like behavior of ions, electrons, neutrons, and protons (tunneling and entanglement) [49,50]. Traditionally, physicists assumed that wet conditions, high (biological) temperatures, a low vacuum, and a decoherent state, such as in the cytoplasm, prevent quantum effects [50]. Whether quantum effects (behavior) occur in the cytoplasm is a highly debated topic. For more information, the reader is referred to the review by Matarèse et al. [51].

In a simulation of two neighboring ion channels on a cell membrane, Salari et al. [50] showed that the quantum states of ions (K^+^ in this case) persist for approximately 100 picoseconds within a channel and for 17 to 53 picoseconds outside a channel. Concluded from their experiments, quantum interference of ions is unlikely due to environmental decoherence. One may assume this applies to the cytoplasm, but it will need to be confirmed with in-depth biological experiments. Nevertheless, Salari et al. [50] suggested that matter-wave interference may underlie the selective transport of ions, since interference occurred between ions of a similar size. They also suggested that quantum interference can increase ion transport to 10^8^ ions per second. This is an important finding, since living cells rely on electrical processes (e.g., biochemical reactions or the flow of ions across cellular membranes), as mentioned elsewhere in this review. The transfer of energy within biological systems involves electromagnetic radiation (e.g., photons) and electric field fluctuations (e.g., electron interactions with photons). Cytoplasmic particles are excited and ionized, which may induce vibrations that damage and disrupt the normal functioning of molecules and biological processes [51]. Quantum effects may influence the formation of morphogenetic fields (organization of biological form development) and bioelectric fields (emerging from ion movement across cell membranes) [52].

Frederiksen et al. [53] and Calvillo et al. [54] suggested that quantum effects can indeed exist within biological systems. Quantum fluctuations arising from the non-constant energy of an electron in an atom may lead to stochastic behavior in the cytoplasm, introducing inherent randomness and indeterminacy in DNA repair, enzyme catalysis, and cellular signaling [53,55]. However, the extent to which these quantum effects extend in time and space within living cells remains unknown. Quantum entanglement may facilitate long-distance communication between morphogenetic fields [51] by influencing the behavior of biological molecules and the speed at which intracellular communication occurs [51,56,57]. In a more physical way, bacteria may respond to environmental signals, such as ion gradients and temperature changes, via actin-like proteins [58]. At least 25 bacterial actin homologs have been described [59]. One of these, protein MreB, positioned beneath the inner cell membrane (Figure 1A), facilitates flagellar movement in *Salmonella* and *E. coli* [60], regulates gliding motility in *Myxococcus xanthus* [61], and regulates pili formation in *Pseudomonas aeruginosa* [62]. Bergeron et al. [63] reported unique tubular actin-like structures of unknown function, conserved among the *Verrucomicrobiota* phylum. It would be interesting to determine whether these structures could alter cytoplasmic electrical forces and generate hydrodynamic quantum waves.

Bacteria constantly monitor the physicochemical conditions in their environment and are thus in a permanent state of sentience, i.e., the ability to respond to stimuli and transfer signals via signal transduction pathways to turn target gene expression on or off. *Escherichia coli* uses approximately 10,000 chemoreceptors, both on the cell surface and embedded in the cell membrane (Figure 1A), to maintain sentience. Each receptor has multiple binding sites [64]. Phytochrome receptors (BphP) detect light [65]. Tsr receptors detect L-serine (Tsr), auto-inducer 2 (AI-2) quorum-sensing (QS) molecules, and redox potential [66]. Aspartate/maltose and ribose/galactose are sensed by the receptors Tar and Trg, respectively [67,68,69]. FixL receptors detect the oxidative state of the environment [70].

Sensing the environment stimulates specific signaling pathways that alter gene expression, activation, and cellular behavior. In bacteria, these pathways involve one-component systems (OCSs) and two-component systems (TCSs). OCSs such as LuxI-LuxR are found in several species, and thousands of LuxR-type receptors have been identified [70]. The LuxR protein acts as a transcription factor, and LuxI functions as a synthase that continuously produces the diffusible signaling molecule acyl-homoserine lactone (AHL) [71]. In bacteria lacking the LuxI gene, the LuxR receptors are called LuxR-solos (LuxRs) [70]. These LuxRs recognize AHL produced by other bacteria. Some LuxR-type receptors interact only with inducers from specific bacterial species [70].

The TCS of *Vibrio* species (e.g., *Vibrio harveyi*) consists of a LuxPQ complex with a membrane-bound histidine kinase (HK) receptor. LuxQ in the LuxPQ complex acts as a kinase when cell numbers are low and as a phosphatase when cell numbers increase [70]. At high cell densities, autoinducer-2 (AI-2) accumulates and binds to LuxPQ. This inhibits single-cell behavior, and cells transition to colony behavior [70]. LuxN, a membrane-bound HK TCS in *V. harveyi*, is highly specific and detects AHL signals known as HAI-1. LuxN can switch from a kinase (low cell density) to a phosphatase (high cell density) [72]. Disruption of tight junctions in intestinal epithelial cells (IECs) leads to the secretion of AI-2-like molecules that interact with the bacterial receptor. The AI-2-like molecules bind to the LuxP receptor in bacterial cells. For more information on QS, the reader is referred to the reviews by Dicks [73] and Bridges et al. [74].

Biofilm formation is a directly measurable output of QS behavior discussed above. In Pseudomonas aeruginosa, biofilm development is coupled to LasR/RhlR signaling, and imipenem-resistant isolates exhibit increased biofilm formation and epithelial adhesion in concert with this signaling [75]. Biofilm-forming capacity in P. aeruginosa also varies with virulence and amoxicillin resistance among clinical isolates, showing that biofilm architecture is a stress-responsive, stochastic trait rather than a fixed one [76].

More than 300 transcriptional regulators in *E. coli* control gene networks [77]. A single cell experiences several cytoplasmic states simultaneously and generates competitive coherence, i.e., the reconciliation between the “now” and the “next” gene expressions. Norris [78] described the competition between gene networks as competitive biological coherent states or “qualia” and included in his discussion mRNA, tRNA, rRNA, proteins, ions, lipids, polyphosphates, polyamines, polyhydroxybutyrates, ATP, CTP, and cAMP. This simple description of qualia, however, does not explain the effect of hyperstructures on cytoplasmic (therefore cellular) behavior [79]. Hyperstructures drive complex enzymatic reactions and form QS molecules [73,80] or give rise to quantum-mechanical processes that affect cell behavior [50].

Sentience also implies that cells need to utilize nutritional resources optimally. The flagellar “machinery” in motile bacteria (e.g., *E. coli*, *Salmonella*, *Pseudomonas*, and *Bacillus*) is regulated by molecules that interact with receptors on the cell surface, generating electrochemical signals to the flagellar “motors” embedded in the cell wall [81,82]. In a nutrient-poor environment, the genes encoding flagellin are repressed, and the cells become non-motile. When reintroduced to a nutrient-rich environment, the genes encoding flagellar formation are activated [83,84]. Other species, e.g., *M. xanthus*, use gliding for mobility [61].

Enzymes in a chaotic or stochastic environment bind randomly to substrates, forming stochastic complexes that lead to temporal “jumps” (sudden increases in production) and a reset of enzyme copy numbers [85]. Cells that are stressed alter the rates at which they degrade and aggregate proteins [86]. Cells that survive treatment with antibiotics showed a decrease in proteolytic activity [87,88]. Cells experiencing optimal growth degrade stress-related transcription factors and sigma factors such as RpoS (RNA polymerase sigma factor S). However, when cells are stressed, the active degradation of these factors is halted so that stress-related proteins accumulate and activate stress-resistance networks [89]. This leads to changes in downstream gene expression, which may lead to the formation of persister cells [90] and the formation of viable but not culturable cells [91]. A reduction in protein production during starvation is regulated by the alarmone (p)ppGpp [92]. The authors have shown that the expression of (p)ppGpp differed from cell to cell within a population of *Bacillus subtilis*, suggesting that gene translation differs for each cell. The production of (p)ppGpp leads to an accumulation of RpoS [93]. Nutrient starvation leads to the degradation of inactive ribosomes and rRNA, thereby providing the stressed cells much needed nucleotides and amino acids [94]. Negative feedback mechanisms repress enzyme production, whereas positive feedback mechanisms amplify metabolite production [86,95,96,97,98].

The ability of bacteria to constantly monitor their environment and alter individualistic and collectivistic behavior, coupled with various forms of cytoplasmic dynamics, is a clear indication of sentience. In other words, bacterial sentience derives from the ability to detect physicochemical changes and adapt by altering their cytoplasmic properties. Stochastic/chaotic randomness keeps the cell sentient and safeguards it against sudden, unpredictable environmental changes. The research conducted by Choudhary et al. [10] on *E. coli* cells under oxidative stress has shown that chaos in the cytoplasm is caused by multiple interacting processes, cell cycles, and changes in cell density. The authors have also shown that the level of chaos depends on the strength of the stress response itself. Chaos is best induced by increased gene expression (e.g., increased catalytic activity of scavenging enzymes). This also implies that chaos develops more readily at high growth rates, as it disrupts the equilibrium of the cell population and leads to overcompensation. Choudhary et al. [11] showed that cytoplasmic chaos in *E. coli* arises from rapid gene alterations coupled with cell growth dynamics and cell–cell interactions. The authors showed that gene expression can be shifted from periodic oscillations to chaos on demand. This suggests that sentience, measured in terms of genetic responses to changing environments, derives from chaotic reactions in a dynamic cytoplasm. In a sense, chaotic responses force cells in a population to diversify and adopt a bet-hedging strategy to protect the community against stressors.

## 4. Conclusions

Our limited understanding of cytoplasmic behavior in bacteria complicates our comprehension of bacterial sentience. Bacteria are constantly exposed to stress, which intensifies as cells age, nutrients are depleted, and metabolite levels (intra- and extracellular) change. Changes in cytoplasmic fluidity and dynamics alter the equilibrium of cytoplasmic particles and cellular behavior. By identifying the drivers of cytoplasmic responses, we should be able to predict when the cytoplasm transitions from a chaotic or stochastic to a deterministic (predictable) form. One may ask whether bacteria deliberately alter their environment through physiological/biochemical reactions to stimulate cytoplasmic responses and drive chaotic, random interactions toward normality (a homogeneous state). Could it be that bacteria generate internal chaos to navigate external chaos? Stochastic/chaotic randomness safeguards the cell against sudden, unpredictable environmental changes. Sentience is so embedded in cellular behavior that it must be encoded in the DNA.

## Data Availability

No new data were created or analyzed in this study. Data sharing is not applicable to this article.
